# Seasonal characterization of mercury contamination along the Portuguese coast: human health and environmental risk assessment

**DOI:** 10.1007/s11356-023-29495-5

**Published:** 2023-08-30

**Authors:** Patrícia Gonçalves Cardoso, Hugo Morais, Daniel Crespo, Daniela Tavares, Eduarda Pereira, Miguel Ângelo Pardal

**Affiliations:** 1 CIIMAR/CIMAR—Interdisciplinary Centre for Marine and Environmental Research, University of Porto, Matosinhos, Portugal; 2 LAQV-REQUIMTE - Associated Laboratory for Green Chemistry, University of Aveiro, Campus de Santiago, 3810-193 Aveiro, Portugal; 3 CFE - Centre for Functional Ecology, Department of Life Sciences, University of Coimbra, 3000-456 Coimbra, Portugal

**Keywords:** Metal contamination, Coastal areas, Risk assessment, Seafood consumption, Edible bivalves

## Abstract

A seasonal characterization of mercury (Hg) accumulation in three different estuaries along the Portuguese coast (i.e. Ria de Aveiro, Tagus estuary and Ria Formosa) was done. For that, it was evaluated: (1) Hg concentrations in abiotic (water) and biotic matrices (flora and fauna); (2) the risk of consumption of local seafood species (e.g. bivalves) to human health; and (3) the environmental risk to Hg exposure. During 1 year, water and biological samples were collected during low tide, in each system for Hg quantification. Our findings revealed that total Hg concentrations in surface waters were higher in Ria de Aveiro and Tagus estuary than in Ria Formosa. In Ria de Aveiro, a particular attention should be given in autumn periods, where Hg levels (≈ 100 µg L^−1^) were considered quite high according to European quality parameters. The same was observed for the Tagus estuary during spring time. Regarding macrofauna Hg levels, no clear seasonal trend was observed. Also, total Hg concentrations in edible species (< 0.5 µg. g^−1^ ww) represent no risk for consumption. However, considering the environmental risk, in Ria de Aveiro, there is a moderate risk (RQ > 0.1) in autumn periods, which can be a matter of concern.

**Supplementary Information:**

The online version contains supplementary material available at 10.1007/s11356-023-29495-5.

## Introduction

Mercury (Hg) is a pollutant of worldwide concern due to its persistence in the environment and strong ability to bio-accumulate and magnify throughout the food web, reaching elevated concentrations in biota, such as top predators (e.g. seafood), which can threaten both ecological and human health (Zhang et al. 2012). Both inorganic and organic Hg (methylmercury) exist in the environment, being the latter the most toxic form to biota and lately to humans. Both forms can be transported and dispersed in the water column and accumulated in the sediments. Considering that the benthic fauna is in close contact with sediments, it can be particularly vulnerable (Elliott and Quintino 2007), especially the bivalves (Cardoso et al. 2013a; Oliveira et al. 2016; Gao et al. 2016), among others.

Hg is subject to complexation and reduction with dissolved organic matter and suspended particulate matter in the water column, affecting its speciation, bioavailability and mobility in aquatic systems (Chakraborty and Babu 2015; Chakraborty et al. 2019). All these processes may vary seasonally due to modifications in physical and biological factors, which can affect the methylmercury bioaccumulation (Diaz-Jaramillo et al. 2013). Therefore, defining temporal patterns in mercury biomagnification rates in food webs can contribute to minimize the monitoring efforts, leading to a decrease in human and ecological risk from Hg exposure (Zhang et al. 2012).

Along the Portuguese coast, Ria de Aveiro and confined areas of the Tagus estuary are historically contaminated with mercury from industrial (e.g. chlor-alkali plants) sources (Cardoso et al. 2019). Currently, despite the efforts to minimize Hg sources (by modification of the electrolysis process), historic emissions of Hg were deposited in the sediments of both systems (Canário et al. 2007; Cardoso et al. 2013b) having the potential to affect the respective trophic webs and ultimately the human health (Cesário et al. 2017).

In the south coast of Portugal, Ria Formosa is not a heavily industrialized area, and according to previous records, there is indication that Hg contamination in surface waters has become stable since the 1970s (Bebianno et al. 2019). However, there are few recent records on the system (Coelho et al. 2014b; Bebianno et al. 2019).

The characterization of Hg contamination and bioaccumulation in specific coastal areas/estuaries and/or biotic groups is a common topic in the literature. Nevertheless, there is a lack of information regarding a broader scenario, comparing distinct systems and food webs in a joint work. So, the main goal of the present work was to do a general temporal characterization of Hg levels in three different estuarine ecosystems (i.e. different Hg sources, water basins and estuarine characteristics) and respective trophic webs, along the coast. For that, different ecosystem compartments were analysed (abiotic — water and biotic — primary producers and macrobenthos) in three different coastal areas: Ria de Aveiro, Tagus estuary and Ria Formosa in order to (1) evaluate bioaccumulation along the trophic web; (2) to evaluate the risk of consumption of certain seafood species (e.g. bivalves) to human health; and (3) to evaluate the ecological risk of exposure to Hg contamination.

## Materials and methods

### Study sites

**Fig. 1:**
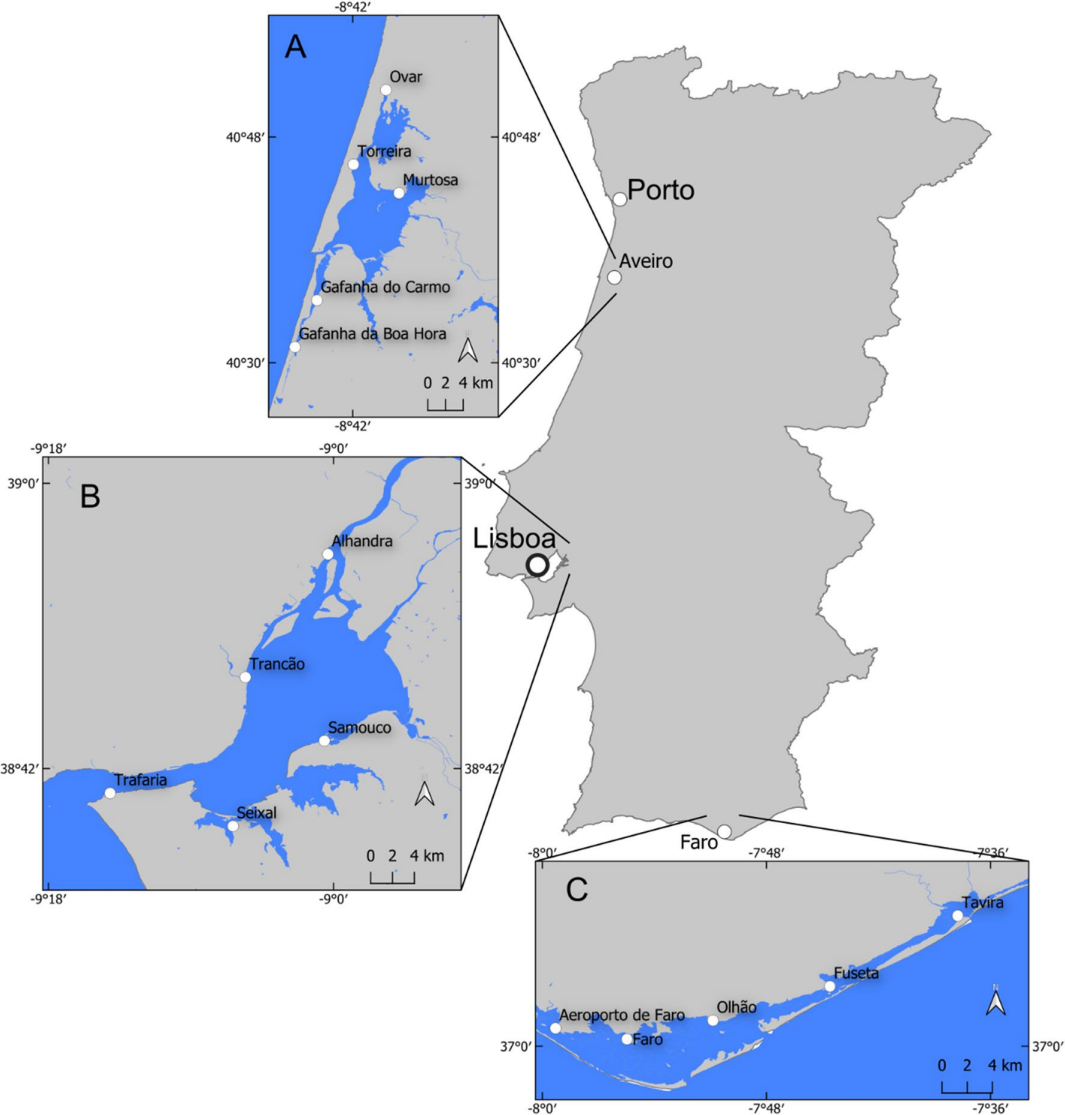
Location of the study sites along the Portuguese coast: (A) Ria de Aveiro; (B) Tagus estuary and (C) Ria Formosa

Sampling was performed in four different periods in three Portuguese estuaries along the coast (Ria de Aveiro: May (sp), July (su), November (au) 2019 and January (wi) 2020; Tagus estuary: March (sp), July (su), October (au) 2019 and February (wi) 2020; Ria Formosa: March (sp), July (su) and October (au) 2019) (Fig. 1). The lack of data in some temporal points in Ria de Aveiro and Ria Formosa was due to some logistic issues related with COVID-19 constraints.

#### Ria de Aveiro

Ria de Aveiro is a shallow coastal lagoon located in the northwestern coast of Portugal (40° 38′N, 8° 45W) with a single connection to the Atlantic Ocean and covering an area of approximately 75 km^2^ (Fig. 1A). It is considered one of the most Hg-contaminated systems in Europe due to continuous discharges of Hg from a chlor-alkali industry between 1950 and 1994 (Pereira et al. 1998). During this period, high concentrations of Hg were deposited in sediments of the Ria. By the way, a recent work from Coelho et al. (2014a) related the observed high suspended particulate Hg concentrations with the resuspension of most contaminated sediment layers.

Five sampling stations (i.e. Ovar, Torreira, Murtosa, Gafanha do Carmo, and Gafanha da Boa Hora) were selected along a transect over the coast (see Fig. 1).

#### Tagus estuary

Tagus estuary is the largest estuary in Portugal, and one of the biggest in Europe, covering an area of approximately 350 km^2^ (Rusu and Guedes Soares 2008) (Fig. 1B). It experienced high levels of Hg contamination as consequence of past industrial activity mainly related with pyrite processing (South margin) and chlor-alkali production (North channel) (Figueres et al. 1985).

Five sampling stations were selected along the estuary, two in the North Margin (Alhandra and Trancão), two in the South margin (Seixal and Samouco) and one in the mouth (Trafaria) (see Fig. 1).

#### Ria Formosa

Ria Formosa is a mesotidal coastal lagoon with 180 km^2^ of area, in permanent connection with the sea through six channels. Located in the South of Portugal, it represents the largest lagoon of the Portuguese coast (Said et al. 2019) (Fig. 1C).

Five sampling stations were selected (i.e. Aeroporto de Faro, Faro, Olhão, Fuseta, and Tavira) along the lagoon (Fig. 1).

### Sampling procedure

At each coastal system, 5 sampling stations were selected (mentioned above) for water collection during low tide and, of those, 3 were also sampled for biota. At each sampling station and occasion, in situ measurements of water temperature, salinity, dissolved oxygen, and pH were taken (multiparameter meter HI 98194). Parallel to the macrobenthic sampling, water from the low intertidal water pools was also collected, in glass bottles (*n* = 3), for analysis of total dissolved Hg.

Macrobenthic samples were collected with a core (141 cm^2^ surface area) to a depth of 20 cm and plants were collected by hand during low tide. Both macrobenthic samples and plants (i.e. different macroalgae, such as *Ulva* sp., *Gracilaria* sp. and *Fucus* sp.) and the seagrass *Zostera noltii* were washed in situ through a 500-µm mesh sieve bag and then placed into plastic bags and transported in a cool box. In the laboratory, organisms were separated in main species and kept in filtered-seawater (adjusted to field salinity) for 24 h in order to eliminate sediment particles. Afterwards, they were separated under a dissecting microscope, identified to the lowest possible taxon, frozen (− 20 °C) and posteriorly freeze-dried for mercury analyses. During low tide, parallel to the macrobenthic sampling, water from the low intertidal pools was also collected for analysis of total dissolved mercury.

The primary producers, mentioned above, were washed to eliminate sediment particles and separated in different organs: roots, stem and leaves. Posteriorly, they were freeze-dried for later Hg analyses.

### Mercury quantification

#### Water

Water samples were filtered through 0.45-µm pore size Millipore cellulose filters and acidified with concentrated nitric acid (HNO_3_ 65%) “mercury free” to pH < 2, as in Sturgeon et al. (1987). Samples were stored in borosilicate glass bottles (according to Sturgeon et al. 1987, there are lower Hg adsorptive losses than in other materials) and maintained in a refrigerated room at 4 °C. Total dissolved Hg analysis in water samples was performed by cold vapour atomic fluorescence spectroscopy (CV-AFS), on a PSA Merlin atomic fluorescence spectroscope coupled with a cold vapour generator, model 10.023, associated with a Merlin PSA detector, model 10.003, and using tin chloride (SnCl_2_ 2% m/v in HCl 10% v/v) as reducing agent. The Hg (II) concentration in water samples was quantified through a calibration curve (*r*^2^ ≥ 0.999) of five standards (0.0 to 100 ng L^−1^) prepared by dilution from the certified standard stock solution of mercury nitrate (998 ± 2 mg L^−1^) in a HNO_3_ solution (2% v/v). For quality control, triplicate samples, blanks and standards were analysed in the sample batch. The detection limit of the method is 1.6 ng L^−1^ and the precision and accuracy expressed, respectively, as relative standard deviation and relative error were < 5%. This analytical methodology is highly sensitive, allowing the measurement of 1 ng L^−1^ of mercury (Mucci et al. 1995).

#### Biota

For total mercury quantification in plants and fauna, freeze-dried samples were analysed in triplicate by thermal decomposition atomic absorption spectrometry with gold amalgamation, using a LECO AMA-254 (Advanced Mercury Analyzer). The detection limit was 0.01 ng. Analytical quality control was performed using certified reference materials (CRMs). For the plants, ERM CD200 was used, while TORT-3 was used on the fauna. The values obtained for the whole CRM analysis ranged from 99 to 150% for the plants and 80 to 112% for the fauna (at 0.05 significance level). Analyses of CRMs were always performed in triplicate and coefficient of variation was lower than 10% for all the analyses.

A summary table of all the species found in the three estuarine systems can be seen in the supplementary material (table S4).

### Risk assessment

#### Health risk

The human health risk associated to the consumption of edible bivalves was assessed through the estimation of the hazard quotient (HQ), which is used to estimate the non-carcinogenic effects of Hg through food ingestion. It is calculated by the ratio between the estimated daily intake (EDI) and the reference risk dose (RfD) of Hg (Eq. 1):

1 $$\mathrm{HQ}=\mathrm{EDI}/\mathrm{RfD}$$

EDI (ug kg^−1^ bw/day) was calculated according to Eq. 2 (Vinceti et al. 2014):

2 $$\mathrm{EDI}=\left[\mathrm{C}\times \mathrm{AvC}\right]/\mathrm{bw}$$

where *C* (μg g^−1^ ww of bivalve) is the mean Hg concentration in the bivalve tissue, AvC is the average consumption of bivalves per day, and bw is the average body weight. The EDI values were compared to the established values of reference doses (RfD), 0.1 μg g^−1^ wet weight of fish for Hg (US-EPA 2017).

For the calculation of EDI, we have converted the total Hg concentration in methylmercury (MeHg), assuming that the proportion of MeHg in the total Hg body burden is higher than 80% (Andersen and Depledge 1997). So, a fraction of 90% of MeHg was considered for all the samples, as in Costa et al. (2020).

HQ < 1 indicate no risk in terms of health effects, while HQ > 1 indicates high probability for long-term health effects (Copat et al. 2012).

Regarding AvC, it was considered an average of 6.8 g day^−1^ of bivalves consumed per capita per day, according to Anacleto et al. (2014).

The mean body weight of Portuguese population was fixed on 70 kg, considering that the average weight in the European population is about 70 kg (Stevens et al. 2012).

#### Environmental risk

For the characterization of the potential risk of a toxic pollutant, it was used the index risk quotient (RQ), calculated by Eq. 3:

3 $$\mathrm{RQ}=\mathrm{Ce}/\mathrm{PNEC}$$

where Ce (µg L^−1^) is the environmental concentration in surface waters and PNEC is the predicted no-effect concentration.

In the present study, it was decided to use the PNEC value (0.39 µg L^−1^) for Hg(II) in the aqueous phase, calculated by Du et al. (2015).

According to Tulcan et al. (2021), values higher than 1 represent a high risk, values below 0.1 represent a low risk, and values between 0.1 and 1 represent moderate risks.

### Data analysis

One-way (factor: season) analysis of variance (ANOVA) on ranks were applied to assess statistical differences in total Hg concentrations in surface waters and biota for each ecosystem, separately. All data were previously checked for normality using the Kolmogorov–Smirnov test and for homogeneity of variances using Levene’s test (Zar 1999). These analyses were done using Statistica 7 software.

Consumption risk assessment was evaluated for the general population, according to the method described in Vinceti et al. (2014).

Biomagnification factors (BMFs) were calculated for selected prey-predator scenarios as BMF = Metal Predator/Metal Prey where Metal Predator and Metal Prey are the concentrations of mercury in micrograms per gram wet weight of the predator and prey, respectively (adapted from Hoekstra et al. 2003). For invertebrates and green macroalgae (Ulva sp.), trace element concentrations of total body homogenates were used for calculations. Prey-predator relationships were defined based on the information from Cardoso et al. (2014). In addition, this information was complemented with information from WoRMS database (www.marinespecies.org).

## Results

### Physicochemical characterization and total dissolved mercury

A physicochemical characterization of each ecosystem was done and the results can be seen in the supplementary material (tables S1-S3).

**Fig. 2:**
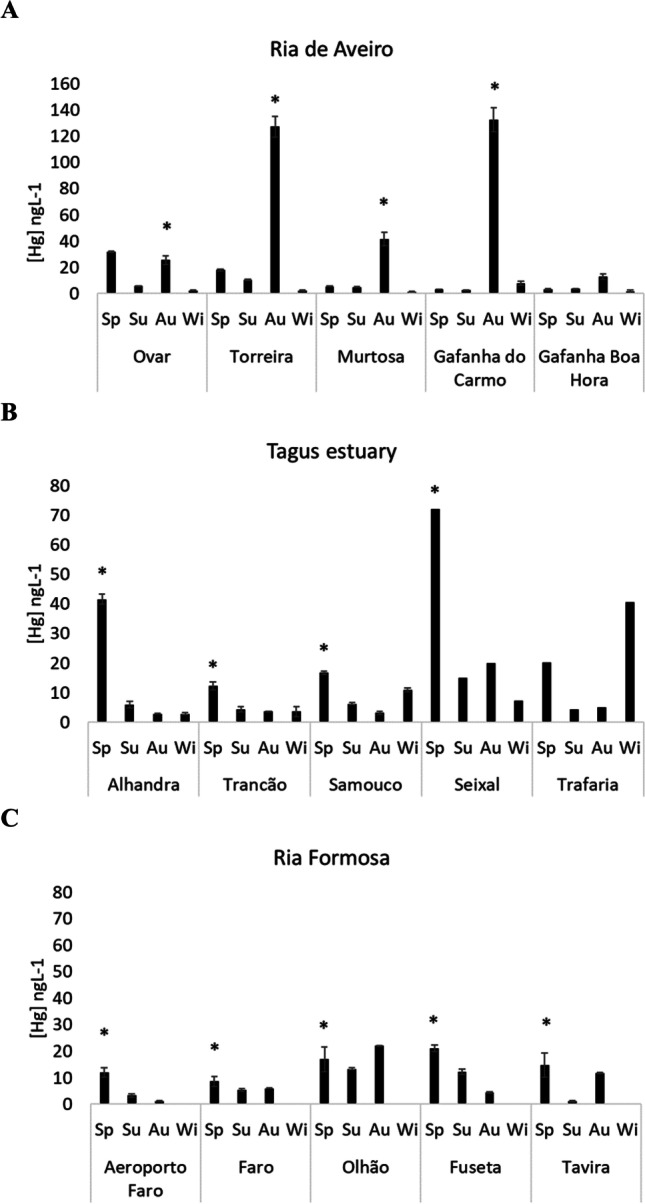
Total dissolved mercury concentrations (ng L^−1^) in surface waters of the three study sites. **A**) Ria de Aveiro; **B**) Tagus estuary; **C**) Ria Formosa. An asterisk indicates significant differences among sites. Sp, spring; Su, summer; Au, autumn; Wi, winter

Total dissolved Hg concentrations were generally higher in Ria de Aveiro (22.2 ± 38 ng L^−1^) than in Tagus estuary (14.9 ± 17.7 ng L^−1^) and Ria Formosa (10.3 ± 6.7 ng L^−1^). In Ria de Aveiro, the highest concentrations were recorded in Torreira (127.3 ± 7.9 ng L^−1^), Gafanha do Carmo (132.6 ± 9.2 ng L^−1^) and Murtosa (41.6 ± 5.2 ng L^−1^) and there was a significant seasonal variation with higher values during autumn campaigns than in other periods, in all the study areas (1-way ANOVA on ranks, *F* = 14.9, *p* < 0.05) (Fig. 2A).

For the Tagus estuary, the highest concentrations were observed in Alhandra (41.6 ± 7.3 ng L^−1^) and Seixal (72.2 ± 0.5 ng L^−1^) during spring period (1-way ANOVA on ranks, *F* = 6.8, *p* < 0.05) (Fig. 2B).

Ria Formosa was the aquatic system with the lowest average Hg concentrations (10.3 ± 6.7 ng L^−1^) being significantly higher during spring period (14.7 ± 4.7 ng L^−1^) (1-way ANOVA on ranks, *F* = 3.7, *p* < 0.05) (Fig. 2C).

### Flora and fauna

The diversity of the macroalgal and seagrass species, associated to the macrobenthic community, was similar in the three coastal systems. The commonly found species were *Ulva* sp., *Gracilaria* sp., *Fucus* sp. and the seagrass *Zostera noltii*.

**Fig. 3:**
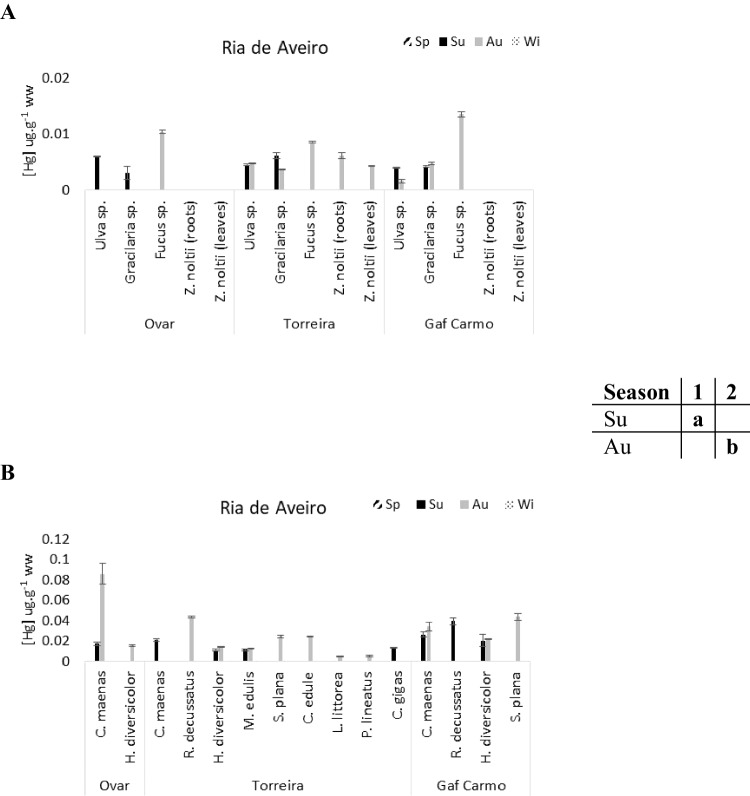
Total mercury concentrations (µg g^−1^ ww) in the flora (**A**) and fauna (**B**) of Ria de Aveiro. The table close to the figure represents the statistical differences (indicated by different letters) between seasons for the plants’ community

**Fig. 4:**
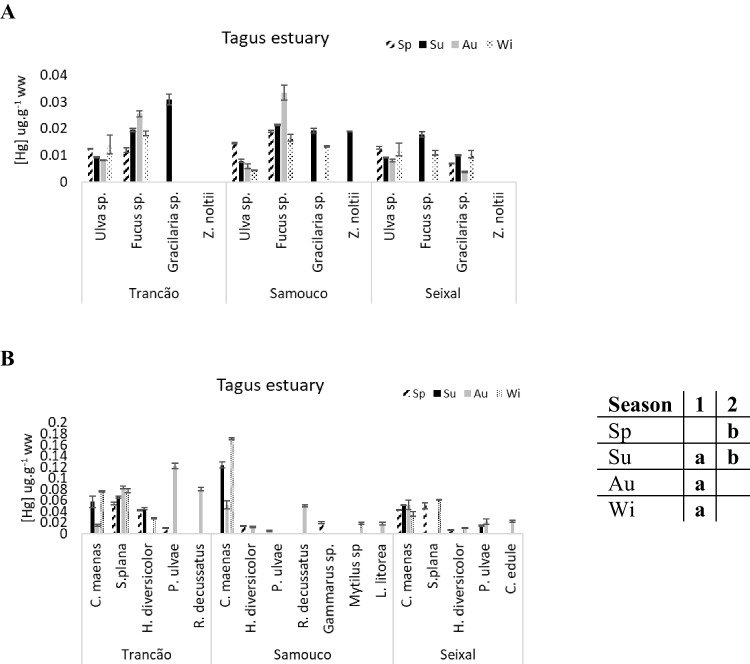
Total mercury concentrations (µg g^−1^ ww) in the flora (**A**) and fauna (**B**) of Tagus estuary. The table close to the figure represents the statistical differences (indicated by different letters) between seasons for the macrofauna community

**Fig. 5:**
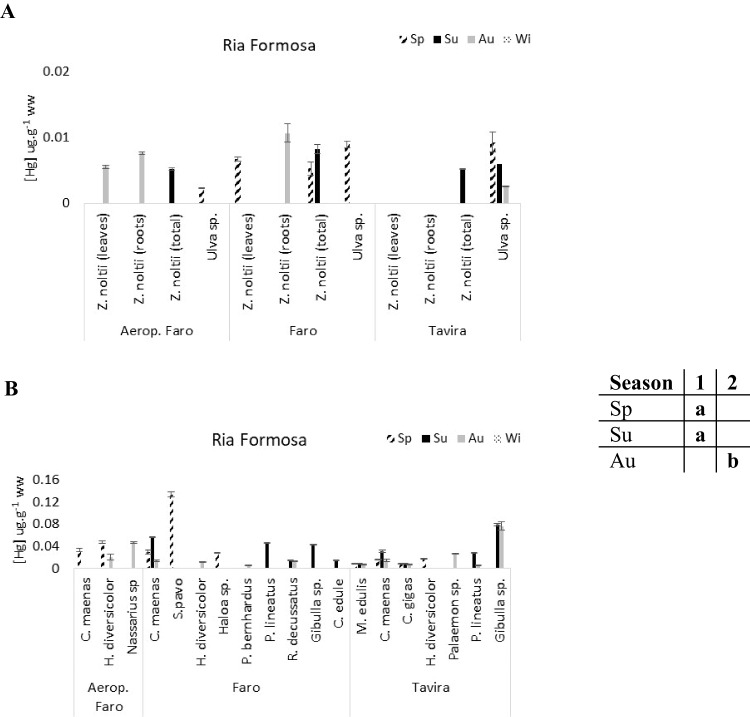
Total mercury concentrations (µg g^−1^ ww) in the flora (**A**) and fauna (**B**) of Ria Formosa. The table close to the figure represents the statistical differences (indicated by different letters) between seasons for the macrofauna community

The average total Hg concentration in the Tagus estuary species (0.014 µg g^−1^ ± 0.007) was clearly higher than in the other two systems (Ria de Aveiro: 0.006 ± 0.003 µg g^−1^, Ria Formosa: 0.006 ± 0.002 µg g^−1^). The same pattern was observed for the macrobenthic communities, having higher mean Hg concentrations in the Tagus estuary (0.047 ± 0.037 µg g^−1^) than in Ria de Aveiro (0.025 ± 0.018 µg g^−1^) and Ria Formosa (0.029 ± 0.027 µg g^−1^) (Figs. 3, 4, and 5).

Most of the macrobenthic species collected in the three systems were the same (e.g. crustacean — *Carcinus maenas*, polychaete — *Hediste diversicolor*, bivalves — *Scrobicularia plana*, *Ruditapes decussatus*, *Cerastoderma edule* and gastropods *— Littorina littorea*, *Peringea ulvae*,) despite in Ria Formosa were identified some particular species (e.g. gastropods — *Nassarius* sp., *Haloa* sp., *Gibulla* sp. and the crustacean — *Pagurus bernhardus*), typical of that system.

A resume table of the list of species found in each estuarine system can be seen in the supplementary material (tables S4-S6).

In a seasonal perspective, there were significant differences in the Hg concentrations in the plants of Ria de Aveiro between summer and autumn periods (1-way ANOVA on ranks, *F* = 4.76, *p* < 0.05) (Fig. 3A). For fauna, no significant differences between sampling periods were observed.

For the Tagus estuary, there were significant differences in the Hg concentrations of the macrobenthic species between seasonal periods. In general, mean Hg values in spring periods were lower than in summer and winter periods (1-way ANOVA on ranks, *F* = 3.27, *p* < 0.05) (Fig. 4B).

In Ria Formosa, the Hg concentrations in the macrobenthic community were significantly higher in spring and summer periods than in autumn (1-way ANOVA on ranks, *F* = 6.87, *p* < 0.05) (Fig. 5B).

### BMFs

The bioaccumulation analysis was restricted to the data available for the trophic webs of the different estuarine systems, since it was just sampled for the macrobenthic community. Based on our results, there is a Hg biomagnification from the lower to the higher trophic levels, which can be seen by the BMF > 1.

In Ria de Aveiro, the BMFs ranged between 1 and 5.4. The highest value corresponded to Hg transfer from *H. diversicolor* to *C. maenas* in Ovar in the autumn period (Table 1).

**Table 1:** Biomagnification factors (BMF) of mercury in the Ria de Aveiro estuarine food web. *Sp*, spring; *Su*, summer; *Au*, autumn; *Wi*, winter; *C. maenas*, *Carcinus maenas*; *H. diversicolor*, *Hediste diversicolor*; *L. littorea*, *Littorina littorea*; *P. lineatus*, *Phorcus lineatus*;* Ulva sp*

(Predators/grazers) (Preys)	*C. maenas* *H. diversicolor* Sp/Su/Au/Wi	*L. littorea* *Ulva* sp. Sp/Su/Au/Wi	*P. lineatus* *Ulva* sp. Sp/Su/Au/Wi
Ovar	-/-/5.40/-		
Torreira	-/1.89/-/-	-/-/1.03/-	-/-/1.18/-
Gaf Carmo	-/1.29/1.59/-		

In the Tagus estuary, BMFs ranged between 0.3 and 14.9. The highest values corresponded to the highest trophic levels, with exception of the Hg transfer from *Ulva* sp. to *P. ulvae* in Trancão in the autumn period that reached a BMF ≈15 (Table 2).

**Table 2:** Biomagnification factors (BMFs) of mercury in the Tagus estuary food web. *Sp*, spring; *Su*, summer; *Au*, autumn; *Wi*, winter; *C. maenas*, *Carcinus maenas*; *H. diversicolor*, *Hediste diversicolor*; *L. littorea*, *Littorina littorea*; *P. ulvae*, *Peringea ulvae*; *Gammarus sp.*;* Ulva sp*

(Predators/grazers) (Preys)	*C. maenas* *H. diversicolor* Sp/Su/Au/Wi	*P. ulvae* *Ulva* sp. Sp/Su/Au/Wi	*Gammarus* sp. *Ulva* sp. Sp/Su/Au/Wi	*L. littorea* *Ulva* sp. Sp/Su/Au/Wi
Trancão	-/1.27/-/2.72	0.92/-/14.85/-		
Samouco	-/-/4.25/-	0.36/-/-/-	1.37/-/-/-	-/-/3.12/-
Seixal	6.50/-/-/3.3	-/1.63/2.76/-		

In Ria Formosa, BMFs varied between 0.7 and 30.3 and curiously the highest values were observed for the grazers *P. lineatus* and *Gibulla* sp. in Tavira in summer and autumn months (Table 3).

**Table 3:** Biomagnification factors (BMF) of mercury in the Ria Formosa food web. *Sp*, spring; *Su*, summer; *Au*, autumn; *Wi*, winter; *C. maenas*, *Carcinus maenas*; *H. diversicolor*, *Hediste diversicolor*; *P. lineatus*, *Phorcus lineatus*; *Haloa sp*; *Gibulla sp.*;* Ulva sp*

(Predators/grazers) (Preys)	*C. maenas* *H. diversicolor* Sp/Su/Au/Wi	*P. lineatus* *Ulva* sp. Sp/Su/Au/Wi	*Gibulla* sp. *Ulva* sp. Sp/Su/Au/Wi	*Haloa* sp. *Ulva* sp. Sp/Su/Au/Wi
Aeroporto Faro	0.70/-/-/-			
Faro	-/-/1.19/-			3.17/-/-/-
Tavira	0.90/-/-/-	-/4.84/2.66/-	-/13.37/30.30/-	

Generally, the highest BMF corresponded to the highest contaminated sites.

### Human health risk assessment and environmental risk assessment

Since HQ was always lower than 1 for all the coastal systems, the health risks for the general population associated to the consumption of bivalves from the three studied systems are reduced. No significant seasonal variations were observed for the three studied areas.

Considering the environmental concentrations of total Hg in surface waters and the PNEC value for inorganic Hg, the RQ values obtained for the different aquatic systems represented low risk for the respective trophic chains, except during autumn in Ria de Aveiro where the highest concentrations in water were found, and which could be classified as moderate risk (RQ = 0.17).

## Discussion

The present work allowed to see that Ria de Aveiro recorded the highest average values of total dissolved Hg in surface waters, even considering study areas located apart from the Laranjo basin, which is considered the most contaminated area in the lagoon (Pereira et al. 2009). For example, in Torreira and Gafanha do Carmo were registered values (≈ 100 ng L^−1^) similar to the ones observed in the most inner area of Laranjo (Cardoso et al. 2014). The highest concentrations were observed during autumn period while in the previous study from Cardoso et al. (2014), the highest values were observed during the summer sampling.

In Tagus estuary, the highest concentrations of dissolved Hg were observed during spring sampling, in Seixal (≈70 ng L^−1^) and Alhandra (≈ 40 ng L^−1^), presenting higher values than in a previous study from Cesário et al. (2018). In the latter study, the highest values recorded in Seixal were in the range of 40–50 ng L^−1^ and in Alhandra ranged between 12 and 24 ng L^−1^. These differences can be justified based on the sampling conditions, since in our study the water samples were collected during low tide while in Cesário et al. (2018), they were collected in flooding or ebbing tide which means that the Hg is more diluted.

Lastly, the Ria Formosa is the least contaminated system reaching the highest values (≈ 20 ng L^−1^) in Olhão during spring sampling. There are very few studies addressing metals contamination in the Ria Formosa. A recent work from Bebianno et al. (2019) only refers that Hg trends in the Ria Formosa water are stable since the 1970s, with no indication of values. A previous work from Coelho et al. (2014b) indicated values of 30–40 ng L^−1^ in the region of Faro (late autumn), which is in accordance with our results.

Comparing the results observed in the three estuarine systems with the environmental quality standards (EQS) (according to the Water Framework Directive), it is possible to highlight that in two sites of Ria de Aveiro (i.e. Torreira and Gafanha do Carmo) and in Tagus estuary (Seixal), during autumn and spring periods, respectively, the values were above the EQS established for mercury (70 ng L^−1^) in surface waters. Also, according to the ecotoxicological assessment criteria (EAC), total dissolved Hg levels in those sites were much higher than the EAC threshold (> 50 ng L^−1^). This means that these areas, in those particular periods, can be more problematic and constitute a matter of concern. In fact, despite the efforts to reduce the Hg sources in the last decades, the Hg accumulated through the years in the sediments of the different estuaries can still be more or less available to the ecosystem depending on physical disturbance (e.g. dredging), or weather events (e.g. increase of temperature or flooding events) which can lead to sediment re-suspension and enhance metal mobilization from the sediments (Coelho et al. 2014a). However, in Ria the Aveiro, the last Hg records (Oliveira et al. 2018, 2022) reveal an ongoing natural recovery of the most contaminated area of Aveiro lagoon associated with natural attenuation by plants, leaching of sediments and through deepening of most contaminated sediments due to the natural sedimentation rates. No signs of any kind of dredging activities in the system were recorded. In the Tagus estuary, even after the end of Hg inputs in the system, the contamination still persists and can spread along the estuary. External factors (e.g. low pH, high organic matter, low dissolved oxygen) can affect Hg methylation in estuarine environments facilitating its spread (Couto and Ribeiro 2022).

Regarding the Hg levels accumulated in flora and fauna of the three systems, the Tagus estuary was the one that, generally, revealed higher Hg concentrations than Ria de Aveiro and Ria Formosa. This can be related to the Hg concentrations in the sediments of respective areas. Since most of the collected species are benthonic, they are in closer association with the sediment than with the water column. In this study, the sediments were not analysed, but by comparing with previous works, the total Hg in sediments of the regions of Trancão and Seixal presented higher concentrations (0.2–1.5 µg g^−1^) (Canário et al. 2005) than those in Ria de Aveiro (e.g. Torreira — < 1 µg g^−1^) (Pereira et al. 2009) or Ria Formosa (0.05–0.1 µg g^−1^) (Coelho et al. 2014b). However, the Hg concentrations found in the macrofauna, inclusive in the edible bivalves, of the three systems were considered low, with values far below the legislation values (0.5 µg g^−1^ ww) established by the European food safety legislation (Commission Regulation 2006).

In a temporal scale, we could observe some significant differences between sampling periods particularly for the macrobenthic community in Tagus and Ria Formosa, but there is not an evident pattern common to all the systems. For example, in Ria Formosa, total Hg concentrations were higher in spring/summer periods than in autumn sampling. Nonetheless, in Ria de Aveiro were not detected seasonal differences. Also, in a previous study from Cardoso et al. (2014), in Ria de Aveiro were not observed clear temporal differences in the Hg content of the studied macrobenthic species and this can be explained based in part on the life span of these species. Since they are long-lived species (i.e. they can live from months to years) and most of the individuals collected were adults (e.g. *S. plana* ≈ 2–3 years; *C. maenas* ≈ 1–2 years; *P*. *ulvae* ≈ 15–20 months) (Verdelhos et al. 2005; Baeta et al. 2005; Cardoso et al. 2002, respectively), they will incorporate Hg in a cumulative way during their life, so it is not dependent on a seasonal variation. This pattern is in part in agreement with the results obtained by Diaz-Jaramillo et al. (2013) for the macroinvertebrates regarding total Hg concentrations. For example, according to the latter study, just in one site were found significantly higher concentrations of total Hg in the summer relative to winter for some of the studied taxa.

However, Gao et al. (2022) found seasonal differences in Hg accumulation in the food web in the coastal waters of Jiangsu (China). According to their findings, Hg concentrations were higher in summer than in spring and autumn. And, the justification for this pattern could be related with the methylation process that tends to be higher in warmer months, favouring the accumulation of the metal in the trophic web. Yet, the difference found between systems can be related to the composition of the trophic web and the food availability that can change seasonally, as well as its Hg content. For example, if a trophic web is constituted mainly by deposit feeders that are in straight connection with the sediment, which is the case of Ria de Aveiro, its Hg content will not change too much along the time. But if the trophic web is more diverse, like in Ria Formosa, and is constituted by different trophic groups including suspension feeders and herbivores, this means that the Hg sources are different (e.g. suspended particulate matter, microalgae, macroalgae), and its content can vary more seasonally, which will influence the entire trophic web. For example, Diaz-Jaramillo et al. (2013) and Reichmuth et al. (2010) observed that in different species of crabs the accumulation of metals was highly variable and often followed environmental concentrations. Also, in the present study, the crab *Carcinus maenas* presented oscillations in its Hg concentrations according to the time of the year.

Concerning the Hg biomagnification through the macrobenthic community, in general, the highest BMF values were related to higher trophic levels, like the predator *C. maenas* and its prey *H. diversicolor* (omnivore). While for lower trophic levels, such as for example, between the green macroalga *Ulva* sp. and the herbivore *P. ulvae* the Hg transfer was lower. This pattern was common for Ria de Aveiro and Tagus estuary and it is corroborated by previous studies (Cardoso et al. 2014). However, for Ria Formosa, the BMF values were quite opposite, since for higher trophic levels there was a Hg biodilution and not biomagnification. This can be related to the fact that the crabs *C. maenas* collected at Ria Formosa during field campaigns belonged to class 1 + (around 30 mm width) (Baeta et al. 2005), which means that they were young individuals, so the accumulation of Hg was lower. Attending that Hg tends to increase with size/age, this can explain some of the results obtained. Also, biodilution of Hg in the macrofauna can be a result of higher organic matter and detritus levels and growth dilution across different trophic levels (Kidd et al. 2012). On the other hand, the higher values obtained in lower trophic levels can be associated to a higher primary productivity, namely production of microalgae, since the species studied are also grazers. Also, the Ria Formosa being a system with warmer waters can be susceptible of higher methylation rates and higher Hg availability to superior trophic levels.

Considering the analysis of risk assessment to the edible bivalve community found in the three ecosystems, the hazard quotients (HQ) were always lower than 1 which means that there is no risk in terms of health effects (Copat et al. 2012). In fact, the concentrations of total Hg found in the biota were quite low, so they do not represent a concern to the human health. On the other hand, the ecological risk assessment highlighted that the high Hg values in surface waters of Ria de Aveiro could have medium risks for aquatic species in specific sites and periods of the year (i.e. autumn periods).

## Conclusions

Our findings demonstrated a clear seasonal trend in Hg concentrations in the water column that can be variable according to the system. Ria de Aveiro and Tagus estuary presented the highest Hg concentrations. In some particular sites of Ria de Aveiro, even far from the most contaminated area of Laranjo, still present quite high Hg values in certain periods of the year.

For the macrofauna species (in particular the bivalves), there is no concern relative to their Hg accumulation levels and possible health effects. But, in those particular sites with high dissolved Hg values, there is a medium risk for the aquatic species in certain periods of the year.

### Supplementary Information

Below is the link to the electronic supplementary material.

Supplementary file1 (DOCX 46 KB)

## Data Availability

The authors confirm that the data supporting the findings of this study are available within the article and/or its supplementary materials.
