# Supplementary material for: Seasonal characterization of mercury contamination along the Portuguese coast: human health and environmental risk assessment
Source: Environ Sci Pollut Res Int. 2023 Aug 30;30(45):101121–32. doi: 10.1007/s11356-023-29495-5 (PMC10541821; doi:10.1007/s11356-023-29495-5)
Supplement: Supplementary file 1 — Supplementary file1 (DOCX 46 KB) [file 11356_2023_29495_MOESM1_ESM.docx]

**Supplementary material**

***Seasonal characterization of mercury contamination along the Portuguese Coast: human health and ecological risk assessment***

**Cardoso PG et al**

Table S1 – Seasonal variation of the physicochemical parameters in Ria de Aveiro study sites.

|  |  | **spring** | | | | **summer** | | | | **autumn** | | | | **winter** | | | |
| --- | --- | --- | --- | --- | --- | --- | --- | --- | --- | --- | --- | --- | --- | --- | --- | --- | --- |
|  | **Study areas** | **Temp.**  **(ºC)** | **Oxygen (mgL^-1^)** | **Salinity** | **pH** | **Temp.**  **(ºC)** | **Oxygen (mgL^-1^)** | **Salinity** | **pH** | **Temp.**  **(ºC)** | **Oxygen (mgL^-1^)** | **Salinity** | **pH** | **Temp.**  **(ºC)** | **Oxygen (mgL^-1^)** | **Salinity** | **pH** |
| **Ria de Aveiro** | Ovar | 19.1 | 7.9 | 4.1 | 8.55 | 20.3 | 7.6 | 1.2 | 7.2 | 16.7 | 8.8 | 2.5 | 7.7 | 12.2 | 11.5 | 3.7 | 7.52 |
|  | Torreira | 19.4 | 8.7 | 25.5 | 8.01 | 22.5 | 9 | 28.9 | 8.11 | 16.4 | 12.1 | 18.3 | 8.32 | 13.6 | 11.6 | 22 | 8.06 |
|  | Murtosa | 19.7 | 9.6 | 14.1 | 7.87 | 22.7 | 6.9 | 30.7 | 8.01 | 15.1 | 8.7 | 9.6 | 7.58 | 11.8 | 11.9 | 14.8 | 7.54 |
|  | Gaf. Carmo | 19.7 | 11 | 11.6 | 8.24 | 20.8 | 5.1 | 24.6 | 7.73 | 16.4 | 9.9 | 15 | 7.83 | 12.5 | 12.1 | 24.5 | 7.84 |
|  | Gaf. Boa Hora | 18.9 | 7.3 | 0.8 | 9.17 | 21.9 | 5.6 | 11.5 | 7.89 | 16 | 8.7 | 1 | 7.95 | 11.2 | 12.3 | 1.5 | 7.93 |

Table S2 – Seasonal variation of the physicochemical parameters in Tagus estuary study sites.

|  |  | **spring** | | | | **summer** | | | | **autumn** | | | | **winter** | | | |
| --- | --- | --- | --- | --- | --- | --- | --- | --- | --- | --- | --- | --- | --- | --- | --- | --- | --- |
|  | **Study areas** | **Temp.**  **(ºC)** | **Oxygen (mgL^-1^)** | **Salinity** | **pH** | **Temp.**  **(ºC)** | **Oxygen (mgL^-1^)** | **Salinity** | **pH** | **Temp.**  **(ºC)** | **Oxygen (mgL^-1^)** | **Salinity** | **pH** | **Temp.**  **(ºC)** | **Oxygen (mgL^-1^)** | **Salinity** | **pH** |
| **Tagus estuary** | Alhandra | 17.45 | 10.7 | 2.15 | 8.5 | 25.9 | 7.5 | 3 | 7.8 | 20.6 | 8 | 1.8 | 8.3 | 16.6 | 10.4 | 0.1 | 7.9 |
|  | Trancão | 18.35 | 6.9 | 6.55 | 8.17 | 24.2 | 6.3 | 25.4 | 7.2 | 21.9 | 3.2 | 4 | 7.9 | 16.7 | 9.5 | 1.5 | 8.3 |
|  | Samouco | 16.7 | 11.35 | 29.8 | 8.63 | 25.7 | 6.8 | 30.3 | 7.7 | 19.8 | 8.6 | 30.4 | 8.1 | 14.8 | 11 | 22.2 | 8.15 |
|  | Seixal | 16.35 | 9.1 | 30.7 | 8.19 | 26.2 | 5.3 | 29.4 | 7.9 | 19.1 | 7.6 | 30.6 | 7.9 | 15.1 | 10.7 | 26.1 | 7.9 |
|  | Trafaria | 13.8 | 6.3 | 18.4 | 6.68 | 24 | 9.9 | 31.9 | 8.0 | 18.7 | 9.4 | 34 | 8.1 | 15 | 11.2 | 31.2 | 8.0 |

Table S3 – Seasonal variation of the physicochemical parameters in Ria Formosa study sites.

|  |  | **spring** | | | | **summer** | | | | **autumn** | | | | **winter** | | | |
| --- | --- | --- | --- | --- | --- | --- | --- | --- | --- | --- | --- | --- | --- | --- | --- | --- | --- |
|  | **Study areas** | **Temp.**  **(ºC)** | **Oxygen (mgL^-1^)** | **Salinity** | **pH** | **Temp.**  **(ºC)** | **Oxygen (mgL^-1^)** | **Salinity** | **pH** | **Temp.**  **(ºC)** | **Oxygen (mgL^-1^)** | **Salinity** | **pH** | **Temp.**  **(ºC)** | **Oxygen (mgL^-1^)** | **Salinity** | **pH** |
| **Ria Formosa** | Aeroporto | 19.05 | 11.8 | 35.95 | 8.38 | 26.2 | 7.6 | 36.3 | 7.83 | 19.7 | 9.4 | 36.5 | 8.06 | - | - | - | - |
|  | Faro | 17.8 | 10.55 | 35.15 | 8.36 | 28.1 | 6.8 | 35.3 | 7.66 | 19.9 | 8.5 | 34.6 | 8.2 | - | - | - | - |
|  | Olhão | 18.05 | 9.35 | 34.6 | 8.38 | 27.3 | 9 | 35.1 | 8.08 | 20.6 | 8.3 | 34.6 | 8.21 | - | - | - | - |
|  | Fuseta | 18.85 | 11.2 | 31.3 | 8.49 | 27.2 | 8.4 | 34.7 | 7.85 | 20.8 | 8.7 | 34.2 | 8.2 | - | - | - | - |
|  | Tavira | 18.25 | 10.5 | 34 | 8.43 | 27.3 | 8 | 34.7 | 8.34 | 19.1 | 9.3 | 34.5 | 8.26 | - | - | - | - |

Table S4 – list of species found in the three studied estuaries during the study period.

|  | **Ria de Aveiro** | **Tagus estuary** | **Ria Formosa** |
| --- | --- | --- | --- |
| **FLORA**  Green macroalga – *Ulva* sp. | x | x | x |
| Red macroalga – *Gracilaria* sp | x | x |  |
| Brown macroalga – *Fucus* sp. | x | x |  |
| Seagrass - *Zostera noltii* | x | x | x |
| **FAUNA**  **Fish**  *Salaria pavo* |  |  | x |
| **Crustacea**  *Carcinus maenas* | x | x | x |
| *Gammarus* sp. |  | x |  |
| *Palaemon* sp.  Pagurus bernhardus |  |  | x  x |
| **Polychaeta**  *Hediste diversicolor* | x | x | x |
| **Bivalvia**  *Ruditapes decussatus* | x | x | x |
| *Mytilus edulis* | x |  | x |
| *Scrobicularia plana* | x | x |  |
| *Cerastoderma edule* | x | x | x |
| *Crassostrea gigas* | x |  | x |
| **Gastropoda**  *Littorina litorea* | x | x |  |
| *Phorcus lineatus* | x |  | x |
| *Peringea ulvae* |  | x |  |
| *Nassarius* sp. |  |  | x |
| *Gibulla* sp. |  |  | x |
| *Haloa* sp. |  |  | x |
